# Fountain of youth—Targeting autophagy in aging

**DOI:** 10.3389/fnagi.2023.1125739

**Published:** 2023-03-29

**Authors:** Lea Danics, Anna Anoir Abbas, Balázs Kis, Karolina Pircs

**Affiliations:** ^1^Institute of Translational Medicine, Semmelweis University, Budapest, Hungary; ^2^Hungarian Centre of Excellence for Molecular Medicine - Semmelweis University (HCEMM-SU), Neurobiology and Neurodegenerative Diseases Research Group, Budapest, Hungary; ^3^Eötvös Loránd Research Network and Semmelweis University (ELKH-SU), Cerebrovascular and Neurocognitive Disorders Research Group, Budapest, Hungary; ^4^Laboratory of Molecular Neurogenetics, Department of Experimental Medical Science, Wallenberg Neuroscience Center and Lund Stem Cell Center, Lund University, Lund, Sweden

**Keywords:** autophagy, aging, rejuvenation, clinical trial, neurodegenerative diseases, direct reprogramming, autophagy-modifying drugs, disease modeling

## Abstract

As our society ages inexorably, geroscience and research focusing on healthy aging is becoming increasingly urgent. Macroautophagy (referred to as autophagy), a highly conserved process of cellular clearance and rejuvenation has attracted much attention due to its universal role in organismal life and death. Growing evidence points to autophagy process as being one of the key players in the determination of lifespan and health. Autophagy inducing interventions show significant improvement in organismal lifespan demonstrated in several experimental models. In line with this, preclinical models of age-related neurodegenerative diseases demonstrate pathology modulating effect of autophagy induction, implicating its potential to treat such disorders. In humans this specific process seems to be more complex. Recent clinical trials of drugs targeting autophagy point out some beneficial effects for clinical use, although with limited effectiveness, while others fail to show any significant improvement. We propose that using more human-relevant preclinical models for testing drug efficacy would significantly improve clinical trial outcomes. Lastly, the review discusses the available cellular reprogramming techniques used to model neuronal autophagy and neurodegeneration while exploring the existing evidence of autophagy’s role in aging and pathogenesis in human-derived *in vitro* models such as embryonic stem cells (ESCs), induced pluripotent stem cell derived neurons (iPSC-neurons) or induced neurons (iNs).

## Introduction

Overcoming the effects of time has long since been one of the holy grails of science. Extending the human lifespan brings a lot of unforeseen consequences. By 2040, age-related neurodegenerative diseases will become the main cause of morbidity in industrialized countries, followed by cancer (Ferri et al., 2005). The extension of lifespan specifically that of healthy lifespan (healthspan) will have enormous effects on society, in both the quality of life and economical points of view. One of the promising “fountains of youth” is related to autophagy and its effects on age-related changes.

Organisms undergo a variety of changes during the process of aging, which impair cellular quality control via disturbed proteostasis, impaired clearance of macromolecules, persistent cellular senescence, stem cell exhaustion, and telomere shortening. Macroautophagy (further referred to as autophagy) plays a key role in aging by maintaining cellular homeostasis through the degradation of unnecessary or dysfunctional components (Hansen et al., 2018; Tabibzadeh, 2023).

Autophagy is an evolutionarily conserved degradation process where cytoplasmic portions and organelles are sequestered into a double-membrane vesicle, an autophagosome (AP), followed by delivery into a degradative organelle, the lysosome, creating an autolysosome (AL) for breakdown and recycling of the resulting macromolecules (Klionsky et al., 2016). This highly dynamic, multi-step process characterized as the autophagic flux is the entire autophagic pathway, which cells can tune according to their metabolic needs (Klionsky et al., 2016).

The autophagic process is mediated by several protein complexes. The most important factors during initiation are the ULK1/2 (Unc-51 like autophagy activating kinase) initiation complex, BECN1 (Beclin1) and some key ATG (autophagy-related-genes) proteins. The main mediator of AP formation is the MAP1LC3B (Microtubule Associated Protein 1 Light Chain 3 Beta; further referred to as LC3B) complex, while SQSTM1 (Sequestosome 1; further referred to as p62) plays role in cargo engulfment (Klionsky et al., 2016; Hansen et al., 2018). In AP-lysosome fusion LAMP proteins (LAMP1 and 2; Lysosomal-associated membrane proteins) play a central role promoting AL formation and degradation.

Autophagy is a critical cellular process for preserving cellular homeostasis by clearance of debris and turnover of cellular compartments. The absence of essential autophagy genes or proteins, as well as impairments in their function cause perturbations in the autophagic flux leading to accumulation of certain autophagic structures and insufficient cellular clearance. Autophagic flux impairment is present in several human neurodegenerative diseases (Zhang et al., 2013).

Regulation of autophagy involves numerous interconnected signaling pathways. Main negative autophagy regulators of autophagy are: (i) the mTOR (mammalian target of Rapamycin), (ii) the PI3K/Akt (Phosphoinositide 3-kinase/Serine-threonine protein kinase), and (iii) the MAPK/ERK (Mitogen-activated protein kinase/Extracellular signal-regulated kinase) signaling pathways (Hansen et al., 2018). mTOR indirectly inhibits autophagy via reducing autophagic protein and lipid synthesis by suppressing transcription factor EB (TFEB) a key regulator of lysosomal biogenesis and autophagy (Hansen et al., 2018; Nnah et al., 2019). The MAPK/ERK signaling inhibits autophagy through enhancing mTOR activity, while the PI3K/Akt signaling blocks autophagy initiation via inhibiting the ULK1 complex (Zachari and Ganley, 2017). Major positive regulators of autophagy are: TFEB, regulating protein and lipid synthesis for autophagy-endolysosomal pathways, Adenosine-monophosphate activated-protein kinase (AMPK), and c-Jun N-terminal kinase (JNK), promoting the ULK1 complex and BECN1, respectively (Dhanasekaran and Reddy, 2017; Nnah et al., 2019; Wang et al., 2020).

**FIGURE 1:**
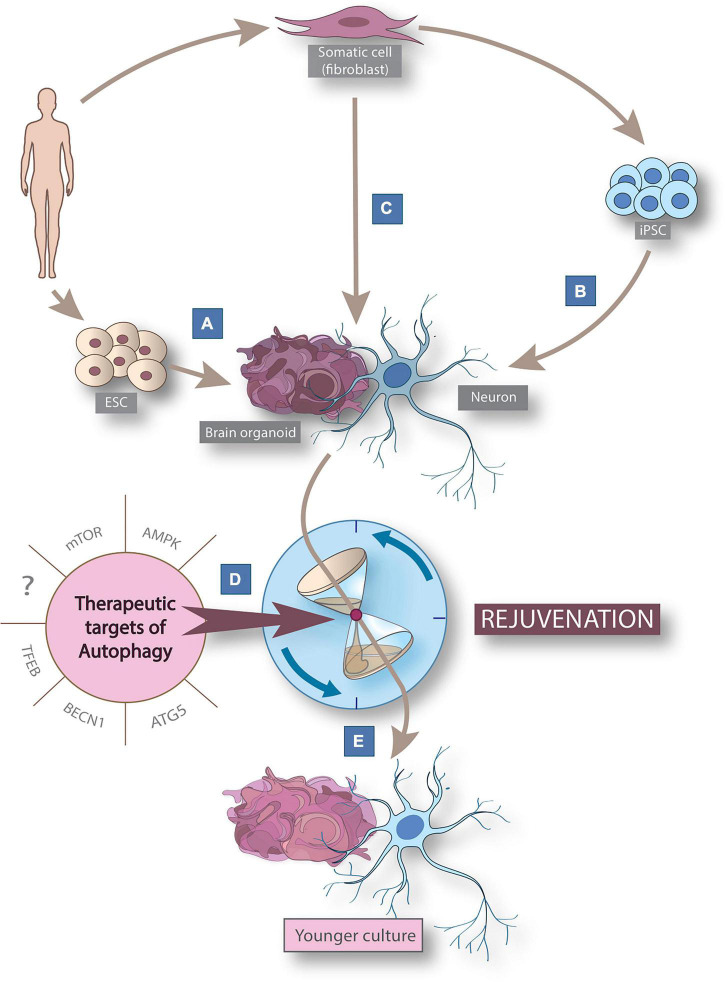
Cellular reprogramming models derived from human somatic cells to study aging and rejuvenation via autophagy modulation. Schematic figure summarizing cellular reprogramming models derived from human somatic cells to study neuronal rejuvenation via autophagy modulation. Human-derived *in vitro* 2D neuronal models can be generated from embryonic stem cells (ESCs) **(A)** or by post-differentiation reprogramming **(B)** from pluripotent stem cells (iPSCs) derived from somatic human cells. In addition, direct reprogramming **(C)** allows the generation of induced neurons (iNs). 3D brain organoids can be generated by 3 routes: from ESCs, iPSCs and iNs. The use of known (mTOR, AMPK, ATG5, BECN1, and TFEB) and yet undiscovered therapeutic autophagy targets **(D)** allows the rejuvenation **(E)** of neuron and brain organoid models for modeling neuronal aging. AMPK, AMP-activated protein kinase; BECN1, Beclin-1; ESC, embryonic stem cell; FOXO, forkhead box class o family member protein; iPSC, induced pluripotent stem cell; mTOR, mammalian target of rapamycin; TFEB, transcription factor EB.

Several studies indicated that activation of autophagy can efficiently extend the lifespan of various organisms such as yeast, worms, flies, and mammals (Hansen et al., 2018; Aman et al., 2021; Kaushik et al., 2021). Moreover, stem cell research revealed, that autophagy plays a critical role in maintaining cellular stemness by regulating the mitochondrial content to help cellular adaptation to different metabolic requirements, and by reducing the accumulation of damaged mitochondria and reactive oxygen species (ROS) (Chang, 2020). Cellular stemness represents an extraordinal capability of self-renewal and escaping aging (Zhu et al., 2015). In stem cells the activity of the autophagic process is remarkably elevated which indicates a crucial role for stemness phenotype maintenance (Chang, 2020; Adelipour et al., 2022). Several studies have shown that induction of autophagy can accomplish rejuvenation of quiescent stem cells that can restore age-related molecular and functional features both *in vitro* and *in vivo* (Figures 1D–E; Chen et al., 2009; Zhu et al., 2015; Leeman et al., 2018; Fang et al., 2020; Navarro Negredo et al., 2020; Park et al., 2021; Tan et al., 2021).

Autophagy is not only a key element in lifespan expansion, but growing evidence indicates its pivotal role in age-related neurodegeneration, contributing to the development of late-onset disorders like Alzheimer’s disease (AD), Parkinson’s disease (HD), and Huntington’s disease (HD) (Menzies et al., 2015). Altogether, growing number of evidence indicated autophagy activation as a potentially beneficial therapeutic target in aging and especially in age-related human specific diseases.

In this review we discuss the current clinical trials in which autophagy modulating therapies are applied to improve symptoms of neurodegenerative disorders. In addition, we summarize the available *in vitro* models that can recapitulate some key aspects of human neuronal aging (Figures 1A–C) and emphasize the need for more efficient tools in future neuronal rejuvenation studies targeting autophagy (Figures 1D–E).

## Autophagy-modifying therapies in age-related neurodegenerative diseases

Basal and selective autophagy are both key mechanisms that ensure the clearance of toxic, cytoplasmic pathological agents like misfolded, mislocalized and aggregated proteins that contribute to many age-related neurodegenerative diseases. The number of damaged organelles and proteins increase progressively throughout human life. Autophagy has also been described as a cell survival mechanism with antiapoptotic properties (Noguchi et al., 2020). During aging autophagy declines progressively in human neurons (Aman et al., 2021). Downregulation of autophagy-related genes such as ATG5, ATG7, ATG16 (Lipinski et al., 2010), and BECN1 (Shibata et al., 2006; Lipinski et al., 2010) was shown in human glioblastoma cells and in postmortem brain tissue. The age-dependent reduction in the autophagic process contribute to impaired cellular homeostasis due to imbalance in cellular resources and accumulation of debris (Aman et al., 2021; Kaushik et al., 2021). Age is obviously the main risk factor for several disorders, including neurodegenerative diseases (Hou et al., 2019). Human studies demonstrate that autophagy dysfunction is present in almost all of the age-related neurodegenerative disorders, such as AD (Nixon et al., 2005; Nilsson et al., 2013; Sun et al., 2014), PD (Zhu et al., 2003; Tanji et al., 2011; Moors et al., 2016; Chang et al., 2017), and HD (Sapp et al., 1997; Martinez-Vicente et al., 2010; Pircs et al., 2022), the question about the role of autophagy in disease development and progression arises.

Previously addressed preclinical studies suggest that autophagy activation can potentially prevent or halt age-related neurodegenerative diseases, thus autophagy targeting interventions may provide new therapeutic options for these patients. Here we summarize therapeutic approaches using pharmacological autophagy inducers targeting uncurable age-related neurodegenerative human-specific diseases in currently ongoing or already completed clinical trials from the last five years dating from 2018 (Table 1). We conducted systematic search at ClinicalTrial.gov using key words for AD, PD or HD and sorted out trials in which agents with autophagy modulating properties were administered in monotherapy as a primary intervention in the last five years between 2018 and 2022. We collected the most promising drugs—those that were tested in more than one trial in the last five years—in Table 1.

**TABLE 1:** Autophagy-modifying drugs used in clinical trials targeting age-related neurodegenerative disease.

Disease	Drug	Drug target	Effect on autophagy	Clinical outcome	Completed trials		Ongoing trials			References
					**ID**	**Results posted**	**ID**	**Status**	**Study start**	
AD	Bryostatin	PKC_*E*_	ERK/JNK signaling pathway	improves cognition	NCT02431468	2018	NCT04538066	A	2020	Nelson et al., 2017; Mandic et al., 2022
					NCT03560245	2020				
	Donepezil	AChE inhibitor	BECN1 and LC3B-II induction	Improves cognition, activity and social behavior	NCT00571064	2018	NCT03810794	R	2019	Boada-Rovira et al., 2004; Zhao et al., 2013; Dasuri et al., 2016; Adlimoghaddam et al., 2018; Khuanjing et al., 2021; O’Bryant et al., 2021
					NCT03073876	2019	NCT04730635	R	2021	
					NCT01951118	2020	NCT04661280	R	2022	
					NCT00477659	2021	NCT05078944	R	2022	
	Idalopirdin (Lu AE58054)	5-HT_6_ receptor antagonist	mTOR inhibiton	Delays memory impairment	NCT02079246	2018	NCT05592678	NYR	2023	Meffre et al., 2012; Wilkinson et al., 2014; Atri et al., 2018
					NCT02006641	2018				
					NCT02006654	2022				
	Levetiracetam	SV2A	BECN1 and LC3B-II induction	Improves spatial memory and executive function tasks	NCT01554683	2020	NCT03489044	A	2018	Sen et al., 2021; Vossel et al., 2021; Zheng et al., 2022
							NCT03875638	R	2019	
					NCT02002819	2022	NCT04004702	NYR	2020	
	Memantine	NMDA receptor antagonist	AMPK and VPS34 induction	Improves cognition and behavioral disturbances	NCT02553928	2019	NCT03703856	R	2019	Kishi et al., 2017; Hirano et al., 2019; McShane et al., 2019; Vucicevic et al., 2020
							NCT05063851	R	2021	
	Metformin	AMPK	AMPK activation	Moderate effect on cognitive impairment	NCT00620191	2020	NCT04098666	R	2021	Campbell et al., 2018; Farr et al., 2019
	Pimavanserin	5-HT_2A_ receptor inverse agonist	ULK1, FIP200, ATG101, BECN1, ATG5, and LC3B modulation	Improves psychotic symptoms	NCT03118947	2020				Ballard et al., 2019; Srinivasan et al., 2020; Ramachandran and Srivastava, 2021
					NCT03325556	2021				
	Sargramostim (GM-CSF)	Immune cells	ATG3, ATG7 and GABARAPL2 activation	Improves cognition, Amyloid-ß and tau pathology	NCT01409915	2021	NCT04902703	R	2022	Potter et al., 2021; Abdelmoaty et al., 2022
HD	Pridopidine	SIGMAR1 receptor agonist	Facilitates nucleocytoplasmic transport of TFEB	Neuroprotective, improves chorea, motor functions and cognition	NCT02006472	2021	NCT04556656	A	2020	Lundin et al., 2010; de Yebenes et al., 2011; Karl et al., 2013; Wang et al., 2023
					NCT03019289	2021	NCT04388969	R	2020	
					NCT01306929	2022	NCT05287503	R	2022	
PD	Ambroxol	Raise the level of GCase	Facilitates nuclear transolcation of TFEB	Neuroprotective, improves chorea, motor functions and cognition			NCT04388969	R	2020	McNeill et al., 2014; Choi et al., 2018; Mullin et al., 2020
							NCT05287503	R	2022	
	Atomoxetine	SNRIs	Dose dependent effect on LC3B-II	Improves attention and impulsivity	NCT01738191	2018				Marsh et al., 2009; Hinson et al., 2016; Warner et al., 2018; Corona et al., 2019
					NCT03651856	2018				
	Cannabidiol	CB1 and CB2 receptors inverse agonist	Prevent JNK MAPK activation	Therapeutic effects in non-motor symptoms, neuroprotective effect	NCT02818777	2019	NCT03944447	R	2018	Yang et al., 2014; Crippa et al., 2019; Huestis et al., 2019; Patricio et al., 2020
							NCT03639064	R	2020	
							NCT05106504	R	2021	
	Dexmedetomidine	α_2_-adrenoceptor agonist	PI3K/Akt signaling pathway	May slow progression of decline in deep brain activity			NCT05376761	R	2022	Rozet et al., 2006; Martinez-Simon et al., 2017; Yu et al., 2019; Lombardo et al., 2020; Nakajima et al., 2021
							NCT05197439	NYR	2022	
	Donepezil	AChE inhibitor	BECN1 and LC3B-II induction	Improves cognition	NCT02206620	2019				Aarsland et al., 2002; Ravina et al., 2005; Baik et al., 2021; Baba et al., 2022
					NCT01521117	2021				
	Exenatide	GLP1 receptor agonist	PKA and PI3K/Akt signaling pathways	Improves motor symptoms, neuroprotective			NCT04232969	A	2020	Aviles-Olmos et al., 2014; Foltynie and Aviles-Olmos, 2014; Athauda et al., 2017
							NCT04305002	A	2020	
							NCT04269642	A	2020	
							NCT04154072	A	2020	
	Istradefylline	ADORA2A receptor antagonist	Activation of autophagy through AMPK-lysosome induction	Improves depression or cognitive impairment	NCT02610231	2019	NCT05333549	R	2022	Liu Y. et al., 2016; Torti et al., 2018; Chen and Cunha, 2020; Kanzato et al., 2020
					NCT01968031	2020	NCT05182151	R	2022	
	Pimavanserin	5-HT_2A_ receptor inverse agonist	ULK1, FIP200, ATG101, BECN1, ATG5, and LC3B activation	Improves psychotic symptoms	NCT00550238	2019	NCT03947216	NYR	2020	Hawkins and Berman, 2017; Kitten et al., 2018; Dashtipour et al., 2021
					NCT01518309	2020	NCT05357612	R	2022	
					NCT03482882	2020				
					NCT03325556	2021				
	Pramipexole	D_2_/D_3_ receptor agonist	AMPK activation	Improves motor complications and depressive symptoms	NCT03521635	2021	NCT04249544	R	2019	Biglan and Holloway, 2002; Reichmann et al., 2003; Mo et al., 2016; Gencler et al., 2022

Ongoing clinical trial status: NYR, not yet recruiting; R, recruiting; A, active, not recruiting. 5-HT_2A_, 5-Hydroxytryptamine 2A; 5-HT_6_, 5-Hydroxytryptamine 6; ACh, acetylcholine; AChE, acetylcholinesterase; AD, Alzheimer’s disease; ADORA2A, adenosine receptor A_2A_; AMPK, AMP-activated protein kinase; ApoE4, apolipoprotein E4; BECN1, Beclin-1; CB1 and CB2, cannabinoid receptor 1 and 2; DAG, diacylglycerol; GABA, γ-aminobutyric acid; GABARAPL2, GABA type a receptor associated protein like 2; GCase, glucocerebrosidase; GLP1, glucagon-like peptide-1; GM-CSF, granulocyte-macrophage colony-stimulating factor; GSK3ß, glycogen synthase kinase 3; HD, Huntington’s disease; HMG-CoA, β-hydroxy β-methylglutaryl-CoA; IMPase, inositol monophosphatase; JNK, c-jun N-terminal kinase; LC3 – MAP1LC3B, microtubule-associated proteins 1A/1B light chain 3B; MAPK, mitogen-activated protein kinase; MARCKS, myristoylated alanine-rich c-kinase substrate; mTOR, mammalian target of rapamycin; NMDA, N-methyl-D-aspartate; p62 – SQSTM1, sequestosome-1; PD, Parkinson’s disease; PI3K, phosphoinositide 3-kinases; PKA, protein kinase A; PKC_E_, protein kinase C; SIGMAR1, sigma non-opioid intracellular receptor 1; SNRIs, selecstive norepinephrine reuptake inhibitor; SV2A, synaptic vesicle glycoprotein 2A; TFEB, transcription factor EB; ULK1, Unc-51 like autophagy activating kinase.

## Alzheimer’s disease

Alzheimer’s disease is a neurodegenerative disease characterized by extracellular deposits of amyloid-ß plaques and intracellular neurofibrillary tangles (tauopathies). Repeated unsuccessful attempts to inhibit the formation of abnormal proteins (tau, amyloid-ß) by pharmacological drugs has led researchers to turn toward autophagy-related interventions (Long and Holtzman, 2019; Fu et al., 2021). In the nervous system in particular, the role of autophagy in maintaining protein homeostasis is essential, therefore treatments targeting autophagy offer a promising therapeutic option (He et al., 2012; Karabiyik et al., 2021). Several pharmacological compounds modulating autophagy have shown beneficial effects against various symptoms of AD.

Memantine (NMDA-receptor antagonist) and Donepezil [Acetylcholine-esterase (AChE) inhibitor] are two of the five FDA approved drugs to treat AD affected patients. The autophagy enhancing effects of these agents were recently shown as to contribute to their neuroprotective properties (Dasuri et al., 2016; Hirano et al., 2019). Memantine and Donepezil are still intensively studied autophagy-modulator agents in AD with completed and still ongoing trials from the last five years as specified by ClinicalTrial.gov database (Table 1). Other promising candidates in connection with autophagy, such as Bryostatin, Idalopirdine (Lu AE58054), and Pimavanserin (Meffre et al., 2012; Wilkinson et al., 2014; Nelson et al., 2017; Atri et al., 2018; Ballard et al., 2019; Srinivasan et al., 2020; Ramachandran et al., 2021; Mandic et al., 2022), are still under clinical testing.

Often drugs with other indications exert neuroprotective effects possibly via autophagy-modulation. Levetiracetam an antiepileptic drug inducing BECN1, and LC3B-II expression is currently tested in AD with promising results. In addition, preliminary data from a currently ongoing phase 2 trial report that Levetiracetam can stabilize memory function not just in epileptiform, but in non-seizure AD patients as well (Sen et al., 2021). Metformin, a widely used antidiabetic drug has been shown to act on autophagy via AMPK activation (Farr et al., 2019). Campbell et al. (2018) published a meta-analysis demonstrating that Metformin use in diabetic patients significantly lower the risk of dementia and AD. Sargramostin, an immunomodulator improved cognition and ameliorated Aß- and tau-pathology in AD patients, possibly via autophagy induction (Potter et al., 2021).

## Huntington’s disease

Huntington’s disease is a neurodegenerative disorder with an autosomal-dominant inheritance caused by an expansion of CAG repeats that leads to an abnormal polyglutamine strand in the huntingtin protein (HTT). The therapeutic targets that alleviate protein misfolding or promote clearance of misfolded proteins generally slow the progression of the disease in HD models (Pircs et al., 2018, 2022; Brattås et al., 2021; Schumann-Werner et al., 2021). HD is unique in terms of its decisive relationship with autophagy. Wild-type HTT plays an important role in the regulation of autophagy. Autophagy dysfunction, which is a characteristic of HD, not only impairs the clearance of protein aggregates and non-functioning organelles, but mutant HTT also results in the loss of the beneficial regulatory role of HTT in autophagy (Martin et al., 2015; Luo et al., 2020). According to the ClinicalTrials.gov database there are an increasing number of clinical trials in HD targeting distinct steps of autophagy, such as Pridopidine, Memantine, Metformin, and Rilmenidine.

Multiple clinical trials demonstrated the effectivity of Pridopidine in HD especially motor symptoms improvements. Additionally, long-term, high patient number trials showed improvements in cognitive and functional symptoms (Lundin et al., 2010; de Yebenes et al., 2011; Karl et al., 2013). A phase 3 trial is still ongoing involving early-stage HD patients to investigate Pridopidine’s effect on disease progression (NCT04556656) (Table 1).

Huntington’s disease is a rare disorder with much less registered clinical trials than in AD or PD. Nevertheless, we review some additional autophagy targeting drugs, which did not meet our selection criteria of being tested in more than 1 trial since 2018, but still show promising results.

Memantine is a previously described drug approved for treatment of AD that has also been tested in HD. Previous clinical trials demonstrated the efficacy of Memantine in preventing progression of chorea and motor dysfunctions (Ondo et al., 2007). Some trials suggested that Memantine could also prevent disease progression (Beister et al., 2004; Hjermind et al., 2011). For the past five years, one phase 4 trial has been registered on ClinicalTrial.gov database completed in 2021, with no results published yet (NCT00652457).

Metformin is a type II diabetes drug that inhibits translation of mutant HTT through the MID1/PP2A/mTOR protein complex, thereby preventing its synthesis in Hdh150 mouse models *in vitro* and *in vivo* (Arnoux et al., 2018). A clinical trial started in 2021 using metformin in HD patients is currently in phase 3 and is recruiting patients (NCT04826692).

Imidazoline-1 receptor agonist Rilmenidine is a frequently used antihypertensive agent. Rilmenidine acts as an mTOR-independent autophagy inducer and can attenuate mHTT-related neurotoxicity through this pathway in HD (Rose et al., 2010; Perera et al., 2018, 2021). A two-year open-label study investigated the efficacy of Rilmenidine in mild and moderate HD patients (trial registration: EudraCT number 2009-018119-14) (Underwood et al., 2017). Although the trial faced limitations (low patient number, no placebo arm, open-label) the authors reported a lower rate of generalized brain atrophy, and smaller decline in mental and disease status scores compared to TRACK-HD data (Underwood et al., 2017).

## Parkinson’s disease

Parkinson’s disease is one of the most common neurodegenerative diseases that causes dopamine deficiency through the loss of dopaminergic neurons in the substantia nigra. In postmortem PD brains intracellular α-synuclein protein aggregation can be observed, which leads to the formation of the so-called Lewy bodies (Hartmann, 2004). Mutation in the LRRK2 gene is one of the most frequent cause of late-onset PD. These mutations account for 5–13% of familial PD and 1–5% of idiopathic PD (Rui et al., 2018). In LRRK2 mutation-related PD increased autophagosomal-lysosomal activity can be observed which causes aggregation of accumulating autophagic vesicles and hinder autophagic clearance (Rui et al., 2018). In addition to the abnormal protein aggregates, there is a dysfunctional metabolism resulting from autophagy dysfunction and disruption of the lysosome degradation pathway (Decressac et al., 2013; Poewe et al., 2017; Hou et al., 2020; Drouin-Ouellet et al., 2022). Despite the high incidence of this incurable disease, current therapies can only delay the progression of PD. Potential autophagy-related therapeutic targets for the treatment of PD are BECN1 and TFEB, which regulate autophagy by degradation of cellular compartments through a specific network called CLEAR (Coordinated Lysosomal Expression and Regulation).

According to the ClinicalTrials.gov database, several drugs with autophagy-modulating properties are involved in more than one trial for PD since 2018.

AMPK-inducer Pramipexole (a D_2_/D_3_ receptor agonist) and Istradefyllin (an adenosin receptor A_2A_ antagonist) are already FDA-approved drugs for PD with beneficial effects on depression, motor and cognitive functions (Biglan and Holloway, 2002; Reichmann et al., 2003; Liu Y. et al., 2016; Mo et al., 2016; Torti et al., 2018; Chen and Cunha, 2020; Kanzato et al., 2020; Gencler et al., 2022).

There are certain autophagy modifying drugs used in neurological disorders such as Atomoxetine [used in attention deficit hyperactivity disorder (ADHD)], Dexmedetomidine (used as sedative in schizophrenia and bipolar disorders), and Cannabidiol (FDA approved drug for epilepsy); that also seems to be beneficial in PD by improving both motor (e.g., dyskinesia) and non-motor symptoms (concentration, behavior, psychosis, sleeping, etc.) (Rozet et al., 2006; Marsh et al., 2009; Yang et al., 2014; Hinson et al., 2016; Martinez-Simon et al., 2017; Warner et al., 2018; Crippa et al., 2019; Huestis et al., 2019; Yu et al., 2019; Lombardo et al., 2020; Patricio et al., 2020; Nakajima et al., 2021).

Other promising pharmacotherapeutic agents currently under clinical testing for PD which are used since decades for other indications such as bronchitis, chronic obstructive pulmonary disease (Ambroxol) or type II diabetes and metabolic syndrome (Exenatide) (Aviles-Olmos et al., 2014; Foltynie and Aviles-Olmos, 2014; McNeill et al., 2014; Athauda et al., 2017).

In summary, autophagy is a promising pathway for targeting age-related uncurable neurodegenerative diseases. Despite the promising outlook of autophagy-based treatments in experimental models, the high number of failed clinical trials call for more reliable pre-clinical models that can capture key aspects of human aging. There is an urgent need to develop human relevant models to study physiological and pathophysiological changes of autophagy during aging and neurodegeneration in order to provide clinically effective therapies.

## Cellular reprogramming models to study autophagy

Several studies indicate impaired neuronal autophagy as one of the key elements in neurodegenerative processes (Labbadia and Morimoto, 2015; Aman et al., 2021; Karabiyik et al., 2021). Our current knowledge is mostly based on findings in postmortem and animal models, and while these models are unprecedentedly important in aging research, there is also a pressing need to further investigate neuronal autophagy in preclinical *in vitro* human neuronal models. We review the already established human neuronal models that studied autophagy in aging-related conditions and the potential new models that capture aging and the role of autophagy in it (see Figure 1).

*In vitro* reprogramming of somatic cells allows us to investigate human-derived cells which are rare or hard to access, like neurons (Mertens et al., 2016). The main goal is to use and perform detailed downstream analysis of human-derived cellular model systems with a wide range of techniques [electrophysiology, sequencing, mass spectrometry, Western blot (WB), immunostaining, microscopy etc.] to measure autophagy.

Conventional 2D human-derived induced neuronal stem cell models are derived from either human embryonal stem cells (ESC) (Figure 1A) or human-derived induced pluripotent stem cells (iPSC) (Figure 1B). These models have been used in growing numbers for studying autophagy in several late-onset neurodegenerative diseases and revealed autophagic alterations in connection with neurodegeneration (Prajumwongs et al., 2016; Jungverdorben et al., 2017; Mertens et al., 2018; Kolahdouzmohammadi et al., 2021). ESC-derived neuronal models (Figure 1A) used to study cellular pathogenesis are present almost exclusively in stem cell transplantation experiments for ethical reasons (Singh et al., 2016). On the other hand, since they represent prenatal neural age, they are not suitable for investigating neuronal autophagy in aging (Steg et al., 2021). iPSCs can be generated from adult (ergo aged) somatic cells by forced expression of specific genes responsible for cellular stemness (e.g., POU5F1, SOX2, MYC, KLF4) either with viral transduction, microRNAs or chemical compounds (Ghaedi and Niklason, 2019; Figure 1B). Through this procedure, iPSCs are reprogrammed to an earlier epigenetic stage from which they can be further differentiated into any somatic cell types of the human body preserving the genetic features of the donor (Rando and Chang, 2012; Mertens et al., 2018; Ghaedi and Niklason, 2019). Although, since iPSC-neurons get rejuvenated, predicted as being fetal-like neurons based on their DNA methylation profile (Steg et al., 2021), they are useful tools to study the role of autophagy in rejuvenation or in mutation-driven neuronal diseases, but their use in neuronal aging research is constrained by their rejuvenated phenotype (Mertens et al., 2018).

Most neuronal reprogramming studies which focus on macroautophagy alterations have been done in human iPSC PD models so far. Studies of LRRK2-G2019S mutant iPSC-derived dopaminergic neurons (DANs) revealed compromised autophagic maturation and clearance accompanied by morphological alterations of PD-DANs (Sánchez-Danés et al., 2012; Reinhardt et al., 2013; Su and Qi, 2013; Borgs et al., 2016). Borgs et al. (2016) identified upregulation of LC3B, ATG5 and ATG7 genes by RT-qPCR. Sánchez-Danés et al. (2012) identified significant increase both in LC3B^+^ and p62^+^ autophagic structures by immunostaining analysis and elevated level of LC3B-II protein by WB. These findings were confirmed by the accumulation of autophagic structures using electron microscopy (EM) (Sánchez-Danés et al., 2012). Consequently, Su and Qi also detected increased LC3B-II level by WB and immunostaining and increased lysosomal activity in PD-DANs (Su and Qi, 2013), while Reinhardt and colleagues reported decline in basal autophagic activity observed as decreased LC3B-II protein level but accumulation of autophagic structures by EM (Reinhardt et al., 2013). Interestingly, other PD-related mutations – LRRK2-I2020T (elevated LC3B-II and p62 protein level (Ohta et al., 2015)), GBA1-N370S (increased LC3B-II and LAMP1 protein level (Schöndorf et al., 2014); increased LC3B-II, p62, BECN1, LAMP1 and LAMP2 protein level (Fernandes et al., 2016)), SAC1-R258Q (increased number of WIPI2^+^ autophagic structures by immunostaining (Vanhauwaert et al., 2017))—showed similar detrimental effect on autophagic processes in PD-DANs. Present studies have not implicated autophagy impairments in other mutation-related PD, such as PINK1 or PARK2 mutant iPSC-derived neurons. Heman-Ackah et al. (2017) investigated iPSC neurons derived from PD patients with a special SNCA mutation caused by the multiplication of the α-synuclein (SNCA) gene. They found reduced BECN1 protein levels, while GO analysis of transcriptomic data revealed enrichment for autophagic pathways in SNCA mutant PD-neurons (Heman-Ackah et al., 2017). di Domenico et al. (2019) used an exciting new approach to generate iPSC-DANs and iPSC-astrocytes from the same donors and investigated LRRK2-G2019S mutation-driven PD-related changes in the autophagic course using DAN-astrocyte co-culture experiments. The authors emphasized that PD-astrocytes showed even stronger impairment in autophagic clearance than PD-DANs resulting in more robust α-synuclein accumulation. Moreover, these dysfunctional PD-astrocytes were able to impair normal neural functions of healthy control-derived DANs (di Domenico et al., 2019). In hESC-derived PD models no studies could be found up to date where the autophagic process was investigated in detail.

Significant autophagy dysfunction has been also observed in iPSC models of AD. Lee et al. (2014) and Reddy et al. (2016) reported decreased autophagic flux accompanied by accumulation of autophagic vacuoles in iPSC-derived neurons from AD patients. Lee reported decreased p62 and elevated TFEB, LC3B-II and LAMP1 protein level by WB analysis and accumulation of autophagic vacuoles by EM in Presenilin-1 (PS1) mutant AD-neurons (Lee et al., 2014). The authors considered that increased activity of acid sphingomyelinase presented in PS1 mutants caused the impairment in autophagolysosomal processes (Lee et al., 2014). Reddy et al. investigated PS1-depleted iPSC-derived neurons and found decreased LC3B-II/I ratio and decreased p62 level which showed strong correlation with the decreased promoter activity of Sestrin-2 (mediating oxidative stress rescue and AMPK-mTOR signaling (Liang et al., 2016)) accompanied by increased TFEB phosphorylation and mTOR activity (Reddy et al., 2016). According to the work of Verheyen et al. (2015) the induction of autophagy with Rapamycin or Trehalose in iPSC-derived neurons of AD patients reversed tau pathology. Ubina et al. (2019) studied hESC-derived neurons which were genetically modified to induce amyloid-ß accumulation to mimic AD proteinopathy. The amyloid-ß pathology was concomitant with reduction in LC3B^+^ autophagic structures indicating impairment in the autophagic clearance mechanism of amyloid-ß plaques (Ubina et al., 2019).

Numerous studies have investigated autophagy in HD patients-derived iPSC-neuronal models. Malankhanova et al. (2020) used two approaches to model HD neurons by either generating iPSC-striatal medium spiny neurons (iPSC-MSNs) from human embryonic fibroblasts which were genetically modified with CRISPR/Cas9 insertion of expanded 69 CAG repeat long tract or by generating iPSC-MSNs from HD patient-derived blood mononuclear cells with 47 CAG repeats. Large autophagic vacuoles were reported by transmission electron microscopy (TEM) accompanied by an impaired neural morphology (Malankhanova et al., 2020). Camnasio et al. (2012) detected elevated LC3B-II protein level by WB in HD iPSC-neurons, increased lysosomal activity using LysoTracker assay with flow cytometry and accumulation of lysosomes by immunostaining. Nekrasov et al. (2016) identified accumulation of autophagosomes and morphology impairments in HD patient-derived iPSC-GABA medium spiny-like neurons (hiPSC-GMSLNs) using TEM. These aberrations could be partially or fully restored by autophagy modifying agents, such as Lithium or LY294002 (PI3K inhibitor). Interestingly, treatment of HD iPSC-GMSLNs with EVP4593—which normalizes impaired Ca2^+^-transport—showed beneficial effect on autophagic processes by decreasing the number of autophagosomes (Nekrasov et al., 2016). In recent years, other studies also implicated new targets regulating the autophagic machinery in neurons. Fu and Zhang demonstrated in ESC- and iPSC-neurons that decreased autophagy was associated with increased HIPK3 (Homeodomain-interacting protein kinase 3) level in HD neurons (Fu et al., 2018; Zhang et al., 2022). Functional experiments revealed that HIPK3 activates DAXX (Death domain-associated protein 6), a transcriptional suppressor of autophagic genes (ULK1 and BECN1). Moreover, they identified a positive feedback loop between mHTT and HIPK3 as high level of mHTT induces HIPK3 which in turn inhibits autophagic clearance of mHTT. This process is thought to play an important role in disease progression (Fu et al., 2018; Zhang et al., 2022). Aron and colleges investigated the pathology-improving role of USP12 (Ubiquitin specific peptidase 12) in HD iPSC-neurons (Aron et al., 2018). USP12 increased autophagy via ATG7-dependent way by potentiating LC3B turnover (measured by optical pulse labeling assay) and increased the number of autophagic structures identified by EM. The use of super resolution microscopy and immunoprecipitation assay showed significant colocalization of USP12-p62 and USP12-mHTT-Optineurin (another autophagy receptor). USP12 may promote the degradation of mHTT by delivering mHTT to the autophagosomes (Aron et al., 2018). Guo et al. (2013) implicated the role of p53 and DRP1 induced autophagy-mediated cell death in HD iPSC-neurons which showed higher expression of these proteins. Di Pardo et al. (2017, 2019) showed that sphingosine-1-phophate (S1P) metabolization is impaired in iPSC-neurons derived from HD patients, which can also play a role in autophagy dysfunction as elevated S1P level induce autophagy (Di Pardo et al., 2017; Di Pardo et al., 2019). A recently published paper by Bailus et al. (2021) identified another potential autophagy suppressor FKBP5 in human neural stem cells (hNSC) and iPSC-MSNs. The authors propose that FKBP5 binds to mHTT (colocalization was detected by immunostaining) and induce conformational changes which prevent mHTT from autophagosomal degradation (Bailus et al., 2021). FKBP5 seems to have autophagy modulating effects too, as pharmacological or siRNA inhibition induced autophagy manifested in increased level of LC3B-II, p62 and ULK1 protein level (Bailus et al., 2021).

Autophagy impairment was also present in HD astrocytes. Juopperi et al. (2012) reported that autophagy is dysfunctional in the astrocytes generated from iPSCs of an adult-onset patient with 50 CAG repeats and his daughter with juvenile HD (109 CAG repeats). iPSC-astrocytes of the juvenile HD patient showed even stronger upregulation in LC3B^+^ autophagic structures (immunostaining) which was confirmed by TEM as well (Juopperi et al., 2012).

These initial studies point clearly towards a clear alteration in neuronal autophagy in AD, PD and HD; however, the limitations of iPSC-derived neuronal models mimicking human neuronal aging and age-related neurodegenerative diseases need to be kept in mind. The pluripotent phase during reprogramming allows the iPSC-derived neuronal cells to get rejuvenated, and as a consequence, the cells lose many of their aging signatures including DNA methylation (Horvath, 2013; Frobel et al., 2014), transcriptome profile (Mertens et al., 2015), telomer length (Marion et al., 2009), mitochondrial dysfunction (Suhr et al., 2010) and senescence (Lapasset et al., 2011). The clonal expansion of these cells further causes the loss of the genetic heterogeneity originally present in patient-derived samples which can bias our findings especially in cases of idiopathic disease modeling (Mertens et al., 2018). Moreover, studies demonstrated that in many cases iPSC-derived disease models don’t exhibit disease-associated phenotypes under normal culture condition (Dimos et al., 2008; Soldner et al., 2009; Zhang et al., 2010; HD iPSC Consortium, 2012) only upon introducing interventions to mimic aging-like phenotype (ROS, telomere manipulation, progerin, etc.) (HD iPSC Consortium, 2012; Miller et al., 2013; Vera et al., 2016; Wu et al., 2019; Chao et al., 2021).

There are increasing number of exciting attempts to induce aging in iPSCs. Vera et al. (2016) applied telomerase inhibitor treatment to initiate aging-like processes in iPSCs and iPSC-derived neurons. As a result, they observed age-like features such as shorter telomers, increased ROS, DNA damage, reduced dendrite numbers and reduced proliferation. Treatment with telomerase inhibitor in PD-patient derived iPSC-neurons could only present preliminary PD-like phenotype [tyrosine hydroxylase (TH) loss] (Vera et al., 2016). Miller et al. (2013) and Machiela et al. (2020) induced overexpression of progerin (a truncated form of lamin A), which elevates with aging. Accelerated aging-like phenotype of iPSC-neurons included dendrite degradation, TH loss, mitochondrial dysfunction, and protein aggregates. Stress-induced aging was investigated by Zhu et al. (2019) in PD patient iPSC-derived neural progenitor cells (NPCs). They reported decreased SIRT1 (a histone-deacetylase responsible for stress resistance) expression accompanied by increased level of senescence-associated proteins like P53, P21, and P16 and autophagy dysfunction via ATG acetylation upon irradiation and MPTP (1-Methyl-4-phenyl-1,2,3,6-tetrahydropyridine) treatment (Zhu et al., 2019).

Altogether, these initial studies indicate that iPSC-derived neurons can capture some aging aspects of the donor but it is important to note that iPSC reprogramming strongly boosts autophagy in the cells, as highly active autophagy machinery is one of the hallmarks of cellular stemness meaning that autophagy induction is inevitable in iPSC generation (He et al., 2012; Wang et al., 2013; Tang, 2014).

Considering that aging is an incredibly complex and not yet fully understood process, an *in vitro* model which can preserve the genotype and the aging phenotype of the donor would be beneficiary to study neuronal autophagy during human aging in detail (Torrent et al., 2015; Mertens et al., 2018). Direct cellular reprogramming, a relatively novel technique can potentially overcome some of the limitations of currently well-known and widely used cellular reprogramming techniques in studying neuronal aging *in vitro* (Drouin-Ouellet et al., 2017; Inagaki et al., 2022; Figure 1C). During direct neural reprogramming, cells are being transdifferentiated into induced neurons (iNs) without going through a pluripotent or progenitor phase by using a combination of proneuronal transcription factors, microRNAs, growth factors and chemical compounds. The inevitable benefit of this technique is that the generated cells maintain not just the genetic but also the epigenetic—including many of the aging—signature of the parental cells (Drouin-Ouellet et al., 2017; Mertens et al., 2018; Shrigley et al., 2018). This novel technique allowed the generation of *in vitro* physiologically aged human cells which can recapitulate some key aspects of the donors age, such as DNA methylation, transcriptomic aging, DNA damage, mitochondrial dysfunction, accelerated ROS production and oxidized proteins, impaired proteostasis, cellular compartmentalization defects, altered membrane potential and morphology (Jovičić et al., 2015; Mertens et al., 2015; Yang et al., 2015; Huh et al., 2016; Liu M. et al., 2016; Drouin-Ouellet et al., 2017; Tang et al., 2017; Kim et al., 2018; Shrigley et al., 2018; Victor et al., 2018; Pircs et al., 2022).

Altogether, this novel technique is proved to preserve a highly complex epigenetic phenotype. iNs are thus considered as a more realistic representation of the aging-signature of the human donor as iPSC models. Therefore, using direct neural reprogramming in studying human neuronal aging and age-related neurodegenerative diseases can give us fundamental knowledge about the mechanism of epigenetic aging and its role in neuronal diseases (Mertens et al., 2018; Drouin-Ouellet et al., 2022; Pircs et al., 2022). In our latest paper, we investigated neuronal autophagy disturbances using HD-derived induced neurons (HD-iNs) (Pircs et al., 2022). We demonstrated that patient-derived iNs can recapitulate many aspects of the disease phenotype, like accelerated aging, reduced neuronal morphology and autophagy discrepancies (Pircs et al., 2022). HD-iNs showed enhanced epigenetic age based on DNA methylation assay and transcriptional changes. Disease-like phenotype was also manifested in aberrant morphology of HD-iNs having shorter and thinner neurites compared to age-matched healthy iNs. Global proteomic analysis confirmed by WB revealed significant autophagy impairment in HD-iNs affecting the AMPK pathway. Most interestingly, immunocytochemistry staining of autophagic structures (LC3B, p62, and LAMP1) clearly showed that there is a subcellular, compartment-specific impairment of autophagy in HD-iNs characterized by autophagosome accumulation in the neurites (Pircs et al., 2022). Oh et al. (2022) used HD-derived induced medium spiny neurons (iMSNs) generated from healthy donors, symptomatic (HD-iMSNs) and pre-symptomatic HD (preHD-iMSNs) patients using direct reprogramming. They found remarkable age- and disease-related alterations in chromatin accessibility, decrease in LC3B^+^ and increase in p62^+^ autophagic structures and miR-29b-3p miRNA upregulation in HD-iMSNs compared to the control and preHD-iMSN groups (Oh et al., 2022). Target gene pathway analysis of miR-29b-3p revealed the role of miR-29b-3p in senescence and autophagy (Oh et al., 2022). These findings further support the decisive role of autophagy impairments in HD-related neurodegeneration and accelerated aging. Most recently, Drouin-Ouellet et al. (2022) applied the induced neuronal model to study autophagy in idiopathic PD patient-derived induced neurons (iNs) and induced dopaminergic neurons (iDANs). They reported impaired autophagic activity in PD-iDANs, especially at the early steps of the autophagic process, which was also supported by a downregulation of early autophagy-related genes (Drouin-Ouellet et al., 2022). Consequently, age-dependent accumulation of LC3B^+^, LAMP2^+^, and p62^+^ autophagic structures and phosphorylated a-synuclein could be detected in PD-iDANs (Drouin-Ouellet et al., 2022). Through comparing iDANs with iNs generated from the same PD patient, they could observe neuronal subtype specific autophagy impairments resulting in different neuronal vulnerability of iDANs than iNs, which is in line with the dopaminergic neuronal loss present in the human PD brain (Hartmann, 2004; Drouin-Ouellet et al., 2022). Remarkably, these disease-specific features were not present neither in the parental fibroblasts, nor in iPSC-derived iDANs from the same donor, underlining the importance of the direct reprogramming in idiopathic, late onset neurodegenerative disease modeling (Drouin-Ouellet et al., 2022).

As we have mentioned above, direct neuronal reprogramming-based disease models are great tools to capture the genetic and epigenetic features of aging and age-related neurodegenerative diseases to study them in a human-derived *in vitro* system. The so far published studies underline its capability to reveal age-related and disease-specific neuronal features, which could not be achieved in such complexity in iPSC models. Although, induced neurons also have limitations. Using skin fibroblasts as a cell source for transdifferentiation, skin-specific age-related changes may get carried over into iNs, such as UV irradiation-induced DNA changes; these may bring non-neuronal aging-relevant aspects into the system (Mertens et al., 2018; Pircs et al., 2022). The cell source can also be a limiting factor, as skin biopsy is an invasive procedure necessitating medical contribution and ethical approvals (Vangipuram et al., 2013). From the skin biopsy sample, a certain number of fibroblasts can be separated which need to be expanded for experimental purposes, but high passage number fibroblasts (over 15 passages) tend to lose their transdifferentiation capability (Vangipuram et al., 2013; Pircs et al., 2022). This limitation can be, however, overcome with good cell banking practice. The generation of isogenic controls remain extremely challenging compared to iPSC technology, therefore the selection and size of the cohort becomes very important. However, as the iN technology is much less laborious than the iPSC method, higher number of samples can be studied at a time (Drouin-Ouellet et al., 2017; Pircs et al., 2022).

In summary, conventional 2D culture models are highly suitable for investigating aging and disease phenotype in a simplified, human-origin system using various interventions (starvation, drug administration, viral transduction, CRISPR etc.) and a wide range of techniques for analysis (microscopy, immunostaining, molecular biology techniques, multiomics, etc.). Using 2D neuronal cultures (iPSC-neurons and iNs) we already gained substantial human-relevant knowledge about the role of autophagy processes and impairments in neurons and in neurodegeneration, and this will be surely strengthened in the future.

However, neurons cultivated in monolayer culture clearly miss dimensional and spatial complexity (Duval et al., 2017). Besides the widely used 2D cell culture, in the last decades more and more attempts were made to achieve new cellular model systems which have higher complexity than monolayer cultures. 3D cell culture is a fairly new and fast-developing approach which may bridge the gap between classic *in vitro* and *in vivo* research modalities by combining advantages from both (Lancaster and Knoblich, 2014; Amin and Paşca, 2018). The most important benefits of the 3D neuronal models are complex structure, high accessibility, and easy handling which are suitable for high throughput screening purposes (Lancaster and Knoblich, 2014; Amin and Paşca, 2018).

Due to the novelty of the 3D technique in neural aging and neurodegeneration research, most of the related publications are still focusing on the development and optimization of reproducible, high efficiency and disease-specific 3D neural models (Brawner et al., 2017; Centeno et al., 2018; Brighi et al., 2020). However, few pioneer studies have been published recently, which demonstrated that brain organoid models can be used to study autophagy in human neural cells in a more complex view than in monolayer systems (Ha et al., 2020; Lee et al., 2020; Jarazo et al., 2022). The human-derived 3D models used in these studies were generated from iPSCs, derived from healthy donors and patients with neurodegenerative diseases (e.g., PD (Ha et al., 2020; Jarazo et al., 2022) and Niemann-Pick lysosomal storage disease type C (NPD) (Lee et al., 2020). Determination of the autophagic flux was achieved by transcriptomics (NPD: TFEB, RAB39A, RAB23, VAMP7, VAMP8, SNAP25) (Lee et al., 2020), proteomics (PD: EIF2S1, RRAGC, AKT1, HMGB1, IGFR1, LAMP2, 14-3-3ζ, BIRC7) (Jarazo et al., 2022) and detection of autophagy markers by WB (LC3B-II/I, p62) (Ha et al., 2020; Lee et al., 2020). Abnormal autophagy function in patient-derived organoids could be demonstrated in these 3D models (Ha et al., 2020; Lee et al., 2020; Jarazo et al., 2022). The level of LC3B-II was significantly elevated in PD-organoids (Ha et al., 2020) and NPD-derived organoids (Lee et al., 2020). NPD organoids also showed increased p62 expression (Lee et al., 2020). The authors demonstrated the applicability of 3D models for drug testing as they successfully treated the PD-organoids with 2-hydroxypropyl-ß-cyclodextrin (HP-ß-CD) (Jarazo et al., 2022), and LRRK2 kinase inhibitor PFE-360 (Ha et al., 2020) and used valproic acid (VPA) in the NPD-organoids (Lee et al., 2020). PFE-360 and VPA effectively restored autophagic processes by reducing LC3B-II and p62 levels in PD and NPD patient-derived organoids, respectively, (Ha et al., 2020; Lee et al., 2020). While VPA also enhanced genes involved in autophagy induction (TFEB, RAB39A, RAB23) and fusion (VAMP7, VAMP8, SNAP25) (Lee et al., 2020). With the treatment of PD-organoids with HP-ß-CD, autophagic flux could be restored by modulating autophagy regulator proteins LAMP2, 14-3-3ζ, and BIRC7 (Jarazo et al., 2022).

In summary, these studies demonstrate the potential of 3D neural models to study autophagy in neurodegenerative diseases. However, the investigation of neural aging in 3D models has still not been addressed as iPSC-derived organoids lose the aging signature of the donor (Mertens et al., 2018). Transcriptomic analysis revealed that even four-months old brain organoids (longest cultivation time to date) mimic the transcriptomic profile of a second/third-trimester human fetal tissue (Hartley and Brennand, 2017). An extremely interesting and promising development will be the generation of organoids from aged-iPSCs, or iNs (Figure 1E), which could bypass this problem, however, such models remain to be established.

## Conclusion

There is growing evidence of autophagy as a key factor in neuronal aging and health. The autophagy machinery was first described 60 years ago, and several studies have since then demonstrated a strong correlation between autophagic activity and aging with a conserved presence in a wide range of species (Aman et al., 2021). The gradual decline in autophagic activity during aging suggests autophagy has a defining role in youth and age.

As humans age, autophagy declines progressively in the brain (Shibata et al., 2006; Lipinski et al., 2010). Several recent studies have revealed a pivotal role of autophagy impairment in neurodegenerative diseases, especially in late-onset neuronal disorders like PD, AD and HD (Sapp et al., 1997; Zhu et al., 2003; Nixon et al., 2005; Martinez-Vicente et al., 2010; Tanji et al., 2011; Nilsson et al., 2013; Sun et al., 2014; Moors et al., 2016; Chang et al., 2017; Aman et al., 2021; Pircs et al., 2022). Autophagy dysfunction has proven to be a common feature in these age-related neurodegenerative diseases which implicates its definite role in disease development and progression. In preclinical models, restoration of autophagy showed beneficial effects on disease pathology proposing autophagy dysfunction as a key pathogenic focus, which can serve as potential targets for future therapies (Karabiyik et al., 2021). To demonstrate how patients can benefit from autophagy enhancing therapies, we reviewed all ongoing and completed clinical trials using autophagy modulators for the past five years published since 2018. Several drugs have already shown autophagy modulating properties and demonstrated effectiveness in patients with AD, PD and HD (e.g., Donepezil, Memantine, Pramipexole, Pimavanserine). Almost 20% of the drugs tested for the past five years in AD have autophagy modifying effects, while in PD and HD this percentage is even higher, close to 30% and over 35%, respectively. Drug repurposing represents a surprisingly high number of trials published since 2018. Interestingly, many of these “repurposed” drugs with beneficial effects in neurodegeneration induce autophagy (e.g., antidiabetic drugs Metformin and Exenatide or mucolytic drug Ambroxol shown to be effective in PD). A better understanding of the effect of autophagy induction on these repurposed drugs could be more cost and time- effective and potentially help patients affected by age-related disorders. The reviewed preclinical and clinical trials provide strong evidence of the beneficiary role of autophagy in health and disease. However, a remarkable number of clinical studies using autophagy inducing treatments such as Resveratrol (Berman et al., 2017) or Lithium (Hampel et al., 2009; Forlenza et al., 2011; Devanand et al., 2022), fail to fulfill to provide clinically relevant findings. The high number of failed clinical studies highlight the importance of strengthening our understanding of neuronal autophagy by first choosing more appropriate preclinical models and methods to predict drug efficiency in humans. Currently, most of the drug development and preclinical testing are done in animal models, while many age-related diseases are only affecting humans (Dawson et al., 2018). Although these models are inevitably important, their usage in modeling human brain and neuronal aging as well as human-specific neurodegenerative diseases is limited. A better understanding of neuronal autophagy decline in humans during physiological and pathophysiological conditions could be the key for successful clinical interventions.

We reviewed all currently existing human-derived *in vitro* 2D and 3D neuronal models that are able to capture some aspects of human neuronal aging, thus providing a possibility to study autophagy in human aging and age-related diseases in detail (Figure 1). Cellular reprogramming techniques for human neuronal cell generation *in vitro* have allowed the possibility to study evolutionarily conserved cellular processes. There are, however, only a few publications that focus on the alteration of autophagy during aging and age-related diseases in human cellular reprogrammed neuronal models. Stem cell reprogramming models (ESC and iPSC, Figures 1A, B) are useful tools to study mutation-driven autophagy alterations in neurodegenerative diseases, but their juvenile phenotype limits their use in aging-related research. Direct cellular reprogramming (Figure 1C) allows the generation of neurons through transdifferentiation to preserve the genetic profile also in addition to several aspects of the epigenetic age of the donor (Drouin-Ouellet et al., 2017; Mertens et al., 2018). Patient-derived iNs demonstrate disease-specific features including morphological aberrations and autophagy dysfunction in age-related diseases such as HD or idiopathic PD (Oh et al., 2019; Drouin-Ouellet et al., 2022; Pircs et al., 2022). iNs are suitable for testing autophagy targeting therapeutic approaches in a human-relevant model by performing functional experiments and drug screening (Pircs et al., 2022). iNs provide the first possibility to generate patient-specific and epigenetically aged human neurons. This model paves the way to study human neuronal aging in detail and provides great opportunities to better understand how human neuronal aging occurs. These novel findings will serve as a basis for understanding neuronal rejuvenation and aging. This will allow the development of future therapies that may halt or prevent age-related neurodegenerative diseases.

## Author contributions

LD and BK: writing—original draft and writing—review and editing. AA: visualization, writing—original draft, and writing—review and editing. KP: supervision, writing—original draft, and writing—review and editing. All authors contributed to the article and approved the submitted version.
